# In Vivo Evaluation of 3D-Printed Silica-Based Bioactive Glass Scaffolds for Bone Regeneration

**DOI:** 10.3390/jfb13020074

**Published:** 2022-06-05

**Authors:** Dilshat U. Tulyaganov, Elisa Fiume, Avzal Akbarov, Nigora Ziyadullaeva, Saidazim Murtazaev, Abbas Rahdar, Jonathan Massera, Enrica Verné, Francesco Baino

**Affiliations:** 1 Department of Natural-Mathematical Sciences, Turin Polytechnic University in Tashkent, Tashkent 100095, Uzbekistan; tulyaganovdilshat@gmail.com; 2 Department of Applied Science and Technology (DISAT), Politecnico di Torino, 10129 Turin, Italy; elisa.fiume@polito.it (E.F.); enrica.verne@polito.it (E.V.); 3 Department of Prosthodontics, Tashkent State Dental Institute, Tashkent 100047, Uzbekistan; avzal@rambler.ru (A.A.); nigorazstom@yandex.ru (N.Z.); dr.saidazim@mail.ru (S.M.); 4 Department of Physics, University of Zabol, Zabol 98613-35856, Iran; a.rahdar@uoz.ac.ir; 5 Faculty of Medicine and Health Technology, Tampere University, 33100 Tampere, Finland; jonathan.massera@tuni.fi

**Keywords:** bioactive glass, scaffold, 3D printing, in vivo, osteogenesis, bone tissue engineering

## Abstract

Bioactive glasses are often designed as porous implantable templates in which newly-formed bone can grow in three dimensions (3D). This research work aims to investigate the bone regenerative capability of silicate bioactive glass scaffolds produced by robocasting in comparison with powder and granule-like materials (oxide system: 47.5SiO_2_-10Na_2_O-10K_2_O-10MgO-20CaO-2.5P_2_O_5_, mol.%). Morphological and compositional analyses performed by scanning electron microscopy (SEM), combined with energy dispersive spectroscopy (EDS) after the bioactivity studies in a simulated body fluid (SBF) confirmed the apatite-forming ability of the scaffolds, which is key to allowing bone-bonding in vivo. The scaffolds exhibited a clear osteogenic effect upon implantation in rabbit femur and underwent gradual resorption followed by ossification. Full resorption in favor of new bone growth was achieved within 6 months. Osseous defect healing was accompanied by the formation of mature bone with abundant osteocytes and bone marrow cells. These in vivo results support the scaffold’s suitability for application in bone tissue engineering and show promise for potential translation to clinical assessment.

## 1. Introduction

Regenerating bone is a complex task, which requires a synergic multidisciplinary approach and considerable economic, academic and human resources [1] for the development and optimization of novel three-dimensional (3D) porous scaffolds [2] to be used in bone tissue engineering (BTE) applications [3,4].

From a general viewpoint, scaffold performances rely on a fine balance between the chemical/biological properties of materials and their processing conditions. In particular, the former is responsible for osteogenic/angiogenic potential, bioactivity and dissolution rates, while the latter determines the morphology and arrangement of the 3D macroporous architecture. Post-processing treatments (e.g., sintering and surface functionalization) can be eventually performed to preserve or even improve the characteristics of the original biomaterial [5].

Despite this, achieving a reliable prediction of the actual biological response elicited by scaffolds after implantation is quite hard due to the combination of multiple relevant factors derived from the organization of living tissues. In this regard, in vitro cellular tests could provide a valuable scientific contribution, supported by undeniable ethical advantages. Nevertheless, during in vitro experiments, the response of the living systems to contact or exposure with a biomaterial is inevitably simplified: immune and inflammatory responses, in fact, do not take place, and there is no direct interaction of the device with blood, which could lead to clot formation in vivo. Moreover, most in vitro experiments are still based on bi-dimensional (2D) static cultures, which are unsuitable to reproduce the dynamic conditions which are necessary to properly characterize 3D porous scaffolds [6]. Last but not least, in vitro toxicity levels are usually overestimated due to the shorter lifespan of cultured cells, thus allowing only acute toxicity to be actually evaluated [7].

Hence, the importance of in vivo studies lies in the possibility to understand and evaluate the materials’ performances in the complex physiological environment and represents, still today, a mandatory propaedeutic step before clinical trials [8].

Over the years, 3D scaffolds based on bioactive glasses (BGs) have emerged as suitable templates for cells able to induce desirable proliferation and differentiation pathways in vivo. Compared to traditional powder-based grafts, which mainly provide chemical stimuli to cells, the structural cues generated by the scaffold could enhance multipotent mesenchymal stem cell (MSC) differentiation, osteoblast growth, extracellular matrix (ECM) deposition, and subsequent new bone formation [9]. Moreover, Scaglione et al. [10] demonstrated that the specific arrangement in the 3D space of a graft is directly responsible for the quality of newly-formed tissue, resulting in a different organization of collagen fibers. Thus, a relevant difference has to be expected when comparing scaffold-driven bone healing with the regenerative potential of particulates and granules.

Over the last decades, the processing of glass and glass-ceramic materials by additive manufacturing technologies (AMTs) allowed achieving high process reliability and standardization levels, resulting in the appealing possibility of tailoring scaffold properties addressed to specific clinical needs [11]. Among AMTs, robocasting (usually simply called “3D printing”) has been successfully used to process various bioactive glasses and ceramic materials through a relatively easy extrusion-based approach. In this regard, Dai et al. [12] recently showed that 3D-printed Cu-doped BG scaffolds could promote the migration, attachment and proliferation of BMSCs and hUVECs, stimulating bone tissue regeneration while promoting angiogenic pathways. Analogous results, in terms of the angiogenic properties addressed to bone regeneration, were also obtained by doping BGs with Sr, stimulating new blood vessel formation in critical-sized rat calvarial defects within 8 weeks [13]. In another study, Qi et al. [14] reported the production of mesoporous borosilicate BG scaffolds, showing hierarchical porosity given by an ordered mesoporous texture which was combined with a regular macroporous architecture. In vivo studies revealed a significantly enhanced new bone formation in both the inner and peripheral regions of the scaffolds, even without the incorporation of growth factors or stem cells [14].

Focusing on a translational approach, Abarrategi and coworkers [15] reported the production of custom-made robocast hydroxyapatite (HA) and β-TCP scaffolds, which were functionalized with BMP-2 to provide osteoinductive properties both on ectopic and orthotopic sites. This study demonstrated, above all, the enormous advantages that were derived from scaffold customization according to the defect size and shape, thus facilitating the surgical procedure [15].

Recently, our research team applied robocasting technology to fabricate silicate BG scaffolds based on the multicomponent “47.5B” compositional system; the scaffolds were characterized from structural, morphological and mechanical viewpoints in previous studies [16,17,18]. The material used for scaffold production was also tested in the form of particles both in vitro and in vivo [19], revealing a strong pro-osteogenic potential. In particular, a statistically significant difference in bone formation was observed in rabbits by comparing the control (untreated) and experimental groups: in the latter, 47.5B powders were completely resorbed after 3 months in favor of newly-formed bone tissue, thus confirming the high osteostimulatory potential of the material [19].

In light of these promising findings, the present study aims to complete the characterization of robocast 47.5B BG scaffolds and report the post-implantation results in a rabbit model, which was comparatively discussed using 47.5B glass powders as reference material.

## 2. Materials and Methods

### 2.1. Scaffold Manufacturing

Cylindrical grid-like scaffolds (nominal diameter 4.5 mm, 20 layers) were designed and manufactured by robocasting technology in order to match the bone defect geometry, as recommended by surgeons (see Section 2.3). The multicomponent silica-based 47.5B BG with the composition 47.5SiO_2_-2.5P_2_O_5_-20CaO-10MgO-10Na_2_O-10K_2_O (mol.%) was produced using a traditional melt-quenching route as previously described [20,21,22,23], and used as basic material for the production of the printing ink.

The ink formulation, optimized by means of some preliminary trials, included 65 vol.% of Pluronic^®^ F-127 solution and 35 vol.% of 47.5B solid particles (size ≤ 32 μm). The amount of the components was calculated according to Equation (1):

(1) $$m_{i}=\frac{vol.{\%}_{i\;}\cdot V_{f}}{100}\cdot \frac{1}{\rho_{i}}$$

where *m_i_*, vol.%_i_ and *ρ_i_* refer to the mass, the volumetric percentage and the density of each ink component (*ρ*_*F*127_ = 1.067 g/cm^3^, *ρ*_47.5*B*_ = 2.64 g/cm^3^ [16,17]) and *V_f_* is the final volume considering that the cartridge was filled up to 2/3 of its total volume (*V_f_* = 3 mL).

In order to improve the homogeneity of the ink, solid particles were suspended in the Pluronic-based liquid medium by alternating vigorous mixing (2500 rpm for 60 s, Vibrofix VF1 electronic, Ika-Werk) with immersion in an ice bath (temperature ≤ 4 °C, 30 s) to keep the viscosity low and hinder the formation of bubbles. This mixing and cooling protocol was repeated until homogeneous and bubble-free slurry was obtained (approximately from 5 to 10 times).

A Tabletop-3Dn printer (nScrypt Inc., Orlando, FL, USA) with a basic set-up was used for robocasting. After 30-min rest, the paste was transferred to the printer cartridge and extruded directly onto acetate sheets (Colour Copier and Laser Transparency OHP Film, Folex AG, Seewen, Switzerland) under the following conditions: a nozzle diameter of 250 μm (Nordson EFD, Optimum^®^ SmoothFlow^TM^, Westlake, OH, USA), an extrusion temperature in the range of 20 to 24 °C, a printing speed of 2 mm/s and extrusion pressure within 1.24–1.45 bar. Input parameters were inserted by means of dedicated software (MachineTools 3.0) provided by the printer manufacturer.

After being printed, the scaffolds were left to dry in an incubator at 37 °C for about 48 h in order to guarantee uniform drying over the whole volume, thus minimizing the formation of cracks. After drying, the greens were sintered in air, setting a multistage heating program [24] with a final dwell at 625 °C. Readers can find additional details on the robocasting process [16,17].

### 2.2. Scaffold Characterization

The geometrical dimensions of 47 scaffolds were determined from 3 repeated micro-caliper measurements of lengths *L* and diameters *D*, with a resolution of 1 µm.

The total porosity *ε*_0_ (vol.%) of the scaffolds produced was assessed by the gravimetric method [25] according to Equation (2):

(2) $$\varepsilon_{0}=\left(1-\frac{\rho_{s}}{\rho_{m}}\right)\times 100$$

where *ρ_s_* is the apparent density of the scaffold, calculated as a mass-to-volume ratio, and *ρ_m_* is the density of the bulk material (2.64 g/cm^3^), assessed in a previous study by Archimedes’ principle [17].

Results were expressed in terms of the average value ± standard deviation.

A preliminary bioactivity evaluation was carried out to assess the apatite-forming ability of the scaffolds, which is key to allowing bone regeneration in vivo. Specifically, static and semi-dynamic bioactivity tests carried out at 37 °C in a static and orbital shaker incubator (100 rpm), respectively, were performed.

Simulated body fluid (SBF) was prepared following the protocol reported by Kokubo and Takadama [26], with a mass-to-volume ratio of 1.5 mg/mL, according to a previous study by the Technical Committee 4 (TC04) of the International Commission on Glass (ICG) [27]. The pH value of the testing solution was monitored at each time point. At the end of the experiment, samples were rinsed with distilled water and dried overnight at 37 °C in an incubator. In the static bioactivity tests, the volume of SBF was completely substituted with a fresh solution at 48, 120, 168 and 216 h to simulate the physiological recirculation of body fluids, while there was no refresh planned for the tests performed under mild shaking conditions.

Finally, the morphological and compositional features of the scaffolds after the experiments in SBF were assessed by scanning electron microscopy (SEM) and energy dispersive spectroscopy (EDS) (field-emission SEM equipped with EDS; Supra TM 40, Zeiss, Oberkochen, Germany), using an inspection voltage of 15 kV. The selected samples, after the bioactivity tests, were embedded in epoxy resin, cut by a diamond blade, and polished to inspect the scaffold cross-section under the back-scattering mode.

### 2.3. In Vivo Animal Tests

Surgical procedures and related animal care were carried out following the ethical guidelines and rules of local governmental bodies. The permissions for performing in vivo biocompatibility tests were obtained by the local ethics committee (the Ethical Committee of Uzbekistan, under reference no. 4, dated 26 August 2020) and the Ministry of Health of Uzbekistan (the certificate issued to the Interinstitutional Research Center, Tashkent Medical Academy, Uzbekistan, under reference no. 3, dated 13 January 2020).

Implantation protocols were performed at the Interinstitutional Research Center, Tashkent Medical Academy, Uzbekistan, whilst post-surgery histological analyses were completed at the Faculty of Prosthetic Dentistry, Tashkent State Dental Institute, Uzbekistan.

The study was carried out on sixteen healthy and completely matured 1-year old male Chinchilla rabbits (mass within 2.8–3.0 kg). The animals were segmented into two groups (C = control group, glass powder; E = experimental group, robocast glass scaffold) and the experiment was split into two stages of observation at 3 and 6 months (Table 1).

In order to avoid unnecessary stress and discomfort throughout the experimental period, the test subjects were identified according to the testing period and group and kept in individual cages.

In the present research work, 47.5B silicate glass powder (mean particle size = 16.57 μm) was implanted in the control group, as previously reported [19], and used as reference grafting material.

**Figure 1:**
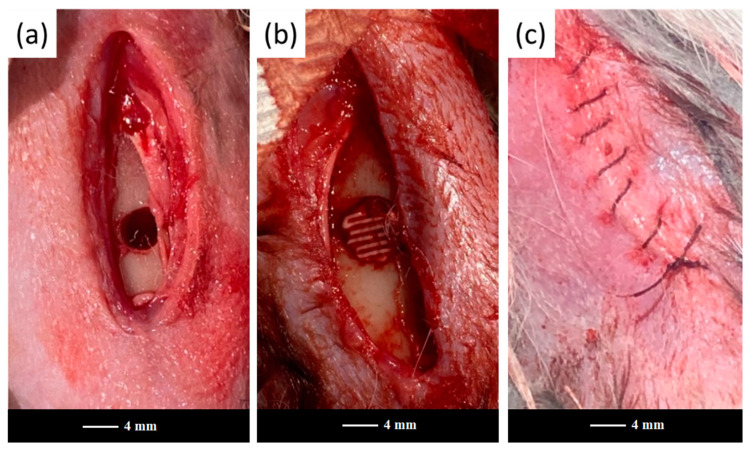
Scaffold implantation in the rabbit’s femoral diaphysis region: (**a**) surgically-created bone defect, (**b**) bone defect filled with a scaffold, (**c**) a wound sutured with catgut.

The 47.5B scaffolds and powders were sterilized in dry heat at 180 °C for 2 h and implanted in the femoral diaphysis region of each animal to fill a previously drilled hole (Figure 1).

After 3 and 6 months of implantation, the rabbits from both the control and experimental groups were sacrificed by immediate decapitation and the femurs were extracted.

**Figure 2:**
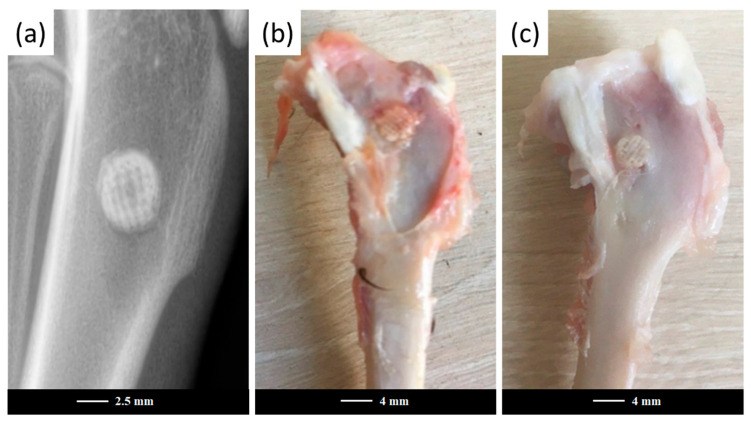
Artificial defects in the experimental group after surgery. X-ray image of the scaffold-filled defect after surgery completion (**a**) and extracted femurs after the cleaning procedure, aimed at removing the surrounding soft tissues after 3 months (**b**) and 6 months (**c**) post-operation. It is clearly visible that the rose-colored part of the scaffold surrounded by soft tissue, just above the drilled hole, remained intact, while nearly-perfect bone-to-scaffold anchoring was appreciable at their interface.

Figure 2 representatively shows the artificial bone defects filled with a robocast grid-like scaffold in the E group after surgery.

All femurs were subsequently fixed in 10% phosphate-buffered formalin for 72 h for histological and histo-morphometrical analyses. Then, the femurs were decalcified in a 4% nitric acid aqueous solution and dehydrated in alcohol. The samples were fixed with paraffin wax and sectioned parallel to the long axis of the femur through the anteromedial aspect of the defect. The cleaned tissue blocks were stained with hematoxylin and eosin (H&E) for histopathological analysis and observed by optical microscopy [28,29].

### 2.4. Statistical Analysis

Statistical analysis was performed by using the Wilcoxon–Mann–Whitney test [30]. The intensity of bone formation for statistical analysis was assessed on the basis of the following histological scoring scale [30,31]: 1 = weak osteogenesis, 2 = medium osteogenesis, 3 = good osteogenesis, and 4 = perfect osteogenesis. Each slide of histopathological sections was divided into four segments for subsequent evaluation, while the average of the scores of the four quadrants represents the score given to the slide [30]. The tests considered the calculation of a statistic named U, whose distribution under the null hypothesis is known [32], and were performed with a level of significance of 5%.

## 3. Results and Discussion

The 3D grid-like architecture of the 47.5B BG scaffolds was analogous to that described in [17]; however, the macroscopic shape was different (cylinder vs. cuboid) to allow a better fitting with the anatomy of the drilled bone defect. As no devitrification occurred upon sintering treatment, the 47.5B scaffolds and powders exhibited comparable microstructures: the XRD pattern, in both cases, was characterized by an amorphous halo centered between 25° and 35° [16,17,24], typical of silica-based glass materials, thus motivating the direct comparison between the two systems.

The geometric features of the scaffolds are summarized in Table 2.

The low values of standard deviation for the geometrical parameters (diameter and length) of the scaffolds reveal a good reproducibility of the samples, as expected for materials fabricated by additive manufacturing technologies. Total porosity (*ε*_0_) can be considered definitely acceptable for the bone tissue engineering scaffolds; In fact, although the resulting *ε*_0_ was very close to the lower threshold recommended (≥50 vol.% [25]), the peculiar grid-like 3D architecture guarantees higher interconnectivity levels within the porous volume

**Figure 3:**
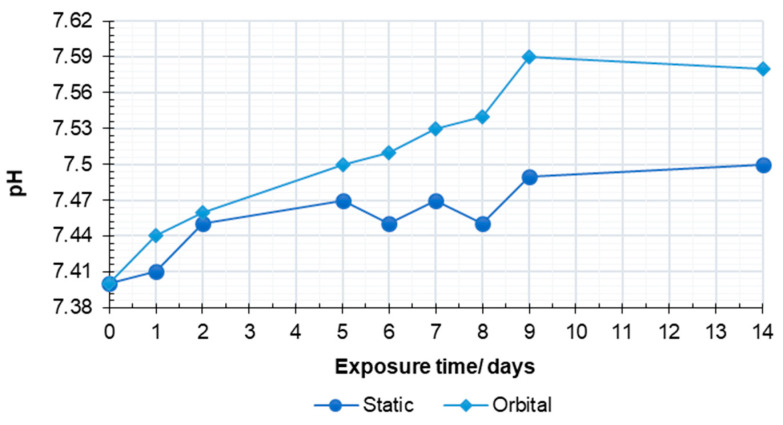
The pH variations of testing solution upon bioactivity assessment under mild shaking and static conditions.

Variations of the solution pH upon the bioactivity tests under mild shaking and static conditions are shown in Figure 3.

Both curves revealed an increasing trend with exposure time, consistent with the glass dissolution/reactivity mechanism that is accepted for bioactive silicate glasses immersed in a simulated physiological environment [33].

Compared to the trend previously observed for the 47.5B glass powder, the curve related to the scaffolds revealed a slower pH increase due to the higher surface area of powders compared to sintered scaffolds [24].

The semi-dynamic conditions in orbital shakers seem to improve the ion exchange mechanisms, leading to higher pH values when compared to static tests. It is known, indeed, that pH changes within the solution are directly related to the dissolution rate of the material [34]. This result is in good agreement with previous studies, reporting a faster bioactivity mechanism under the dynamic dissolution configurations compared to static conditions [35]. This proved a more efficient interaction between the scaffold surface and the solution given by fluid recirculation, which favors the liquid permeation within the scaffold pores, thus involving the whole exposed surface in the ion exchange mechanism. Both the curves reached a plateau after 9 days, indicating an early stabilization of the systems upon exposure to SBF, even under different testing conditions.

**Figure 4:**
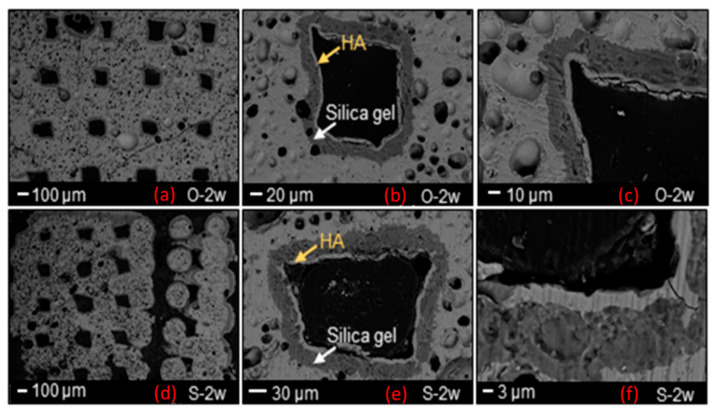
SEM morphological analysis showing the reaction layer formed upon exposure to SBF in an orbital shaker (O-**a**–**c**) and static incubator (S-**d**–**f**) after 2 weeks.

SEM morphological analyses, showing the scaffold cross-section after a 2-week exposure to SBF, are collected and shown in Figure 4. The grid-like architecture of scaffolds is still well distinguishable after the bioactivity experiments.

**Figure 5:**
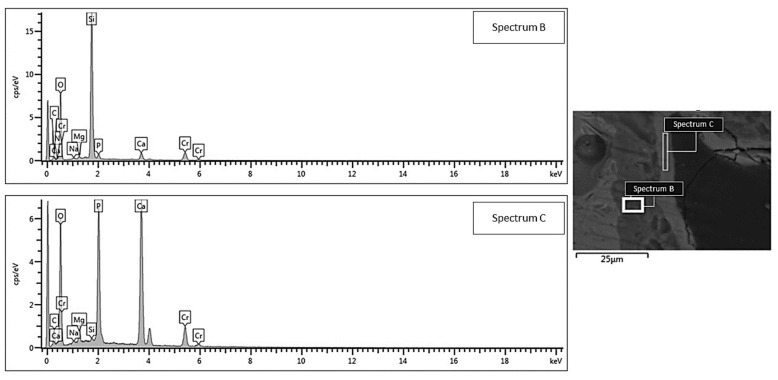
EDS compositional analysis: bi-layered reaction layer formed on the surface of a robocast scaffold subjected to mild shaking in SBF inside an orbital shaker incubator (area B = silica gel, area C = hydroxyapatite).

**Figure 6:**
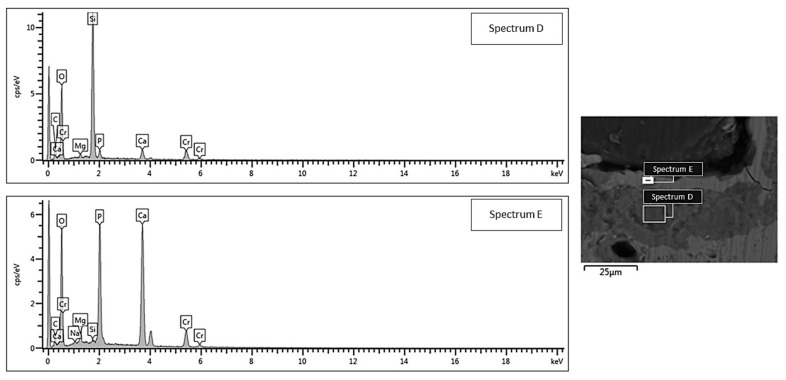
EDS compositional analysis: bi-layered reaction layer formed on the surface of a robocast scaffold subjected to static soaking in SBF inside a traditional incubator (area D = silica gel, area E = hydroxyapatite).

A 30 μm-thick silica gel layer, which typically forms on the surface of BGs upon immersion in SBF [33], was observed on the inner surface of the scaffold pores (white arrow in Figure 4b–e), thus indicating the progressive conversion of the material to a calcium phosphate layer (yellow arrows in Figure 4b–e), as also confirmed by the compositional analysis through EDS (Figure 5 and Figure 6). At the end of the test, the calcium phosphate layer was more homogeneous and continuous in the scaffolds tested under mild shaking conditions, however, the thickness was definitely comparable (≈4–5 μm) in both cases. EDS analyses, performed on the reaction layers upon 14-day exposure to SBF in mild shaking (Figure 5) and static (Figure 6) conditions, confirmed the formation of a reaction bilayer (silica gel + calcium phosphate) with a Ca/P ratio of 1.48 in the silica-rich region and 1.55 on the top surface. The latter value is quite close to the Ca-to-P ratio of stoichiometric HA (1.67) and suggests the formation of Ca-deficient HA, as reported in other previous studies on other silicate BG compositions [36,37,38].

It is universally recognized that the formation of HA in a simulated physiological environment represents a valuable and affordable criterion to preliminarily evaluate the bioactive potential of biomaterials; although, being aware that in vitro conditions can only roughly match those in the human body. The regenerative ability of BG-based scaffolds, in fact, depend on the combination of multiple factors, including the material’s chemical composition (which determines angiogenic potential, bioactivity and dissolution rates), the manufacturing process (which is responsible for pore characteristics, mechanical performances, tissue ingrowth and cell migration) and scaffold post-treatments/functionalization. Because of all these reasons, in vivo tests are necessary to definitely prove the clinical suitability of implantable materials for tissue engineering and regenerative medicine.

Compared to the implantable materials in the form of powders, scaffolds offer the unique possibility of providing additional topographical stimulation to cells as well as a mechanical support for tissue ingrowth and vascularization, thus accelerating the overall healing process.

In recent work, in vitro and in vivo tests were performed in order to assess the biological responses of 47.5B BG powders [19]. In vitro experiments confirmed the positive interaction of 47.5B glass with all the analyzed cellular phenotypes (osteoblastic, endothelial and mesenchymal stem cells), while the in vivo tests revealed the existence of a statistically significant difference between the osteogenesis score in the control and the experimental groups at all the observation periods (1, 2 and 3 months). The high level of osteointegration, as well as the absence of a severe inflammatory response, demonstrated the suitability of 47.5B glass as BTE material, motivating further studies on the use of these bioactive systems for the production of mechanically reliable 3D porous scaffolds [19].

In the present study, osteogenesis levels induced by the presence of the scaffolds were discussed in comparison with the new bone formation observed after the implantation of 47.5B BG in granular form.

All the rabbits survived throughout the implantation stages, exhibiting ordinary behavior with no reports of adverse effects, such as allergies or other immunologic reactions, abscess formation or rejection of the scaffolds. Observation of the histopathological sections did not reveal any inflammation or adverse tissue reaction around the implant. A preliminary examination of tissue blocks by optical microscopy revealed proper healing with no signs of inflammatory infiltrate, degeneration or osteolysis in both the C and E groups.

**Figure 7:**
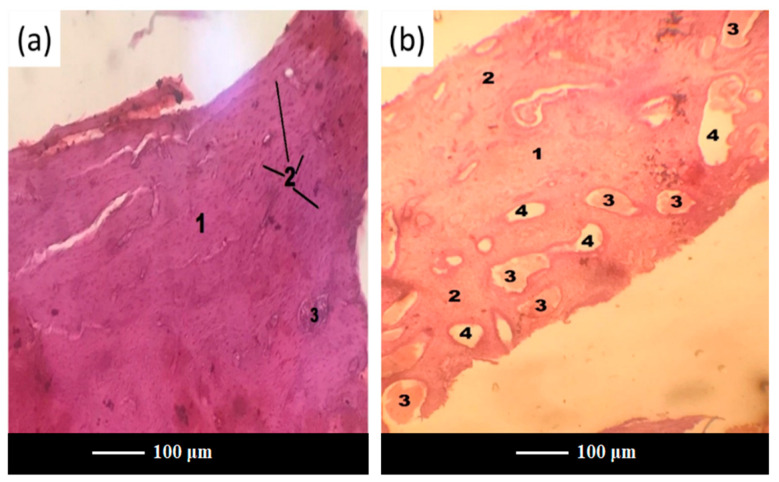
Histopathological sections of the femoral cortical area treated with scaffold (**a**) and glass powder (**b**) after 3 months. Legend: 1—new bone, 2—osteocytes, 3—fat inclusions to yellow bone marrow, 4—the bone marrow vascular niche.

After 3 months of implantation, histomorphometric analyses showed that both scaffolds and glass powders allowed bone ingrowth into the defect (Figure 7a,b), exhibiting a comparable tissue response. In both cases, in fact, the growth of normal healthy trabecular bone, confirmed by the presence of numerous osteocytes in the form of spots, was observed at the implantation site, thus suggesting the gradual resorption of scaffold and glass powder, followed by ossification.

Interestingly, scaffold-treated defects were more densely packed than those treated with glass powder, thus leading to vascular hyperemia at the defect site in group C (Figure 7b). This is an important achievement, considering that one of the major problems in the healing of bone defects is an insufficient or absent blood supply within the defect [39]. Likewise, the presence of sufficient vascularization and a transport system ensures an adequate supply and exchange of nutrients and wastes, which is crucial for supporting cell survival and growth for a longer period as well as for regenerating larger amounts of tissues [40].

**Figure 8:**
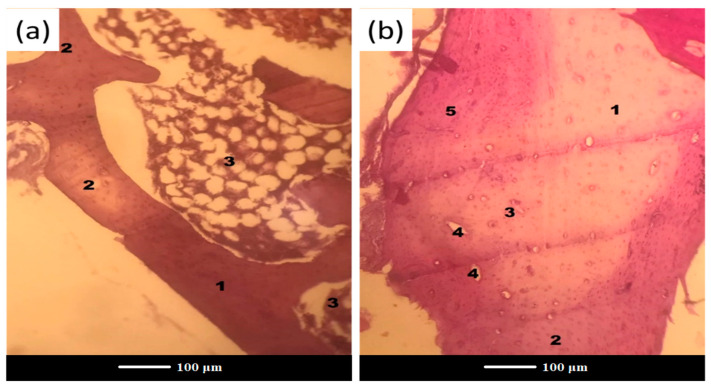
Histopathological sections in the cortical area of the femur treated with (**a**) scaffold and (**b**) glass powder after 6 months. Legend: 1—new bone, 2—osteocytes, 3—fat inclusions to yellow bone marrow, 4—the bone marrow vascular niche, 5—the old bone.

After 6 months of implantation, residues of glass were completely embedded into bone trabeculae, suggesting full resorption and/or osteointegration of the bone grafts in both the C and E groups (Figure 8).

Nevertheless, the most important finding from the histological examinations is that a scaffold-implanted defect showed a more homogenous tissue formation throughout the entire defect when compared to the control.

**Figure 9:**
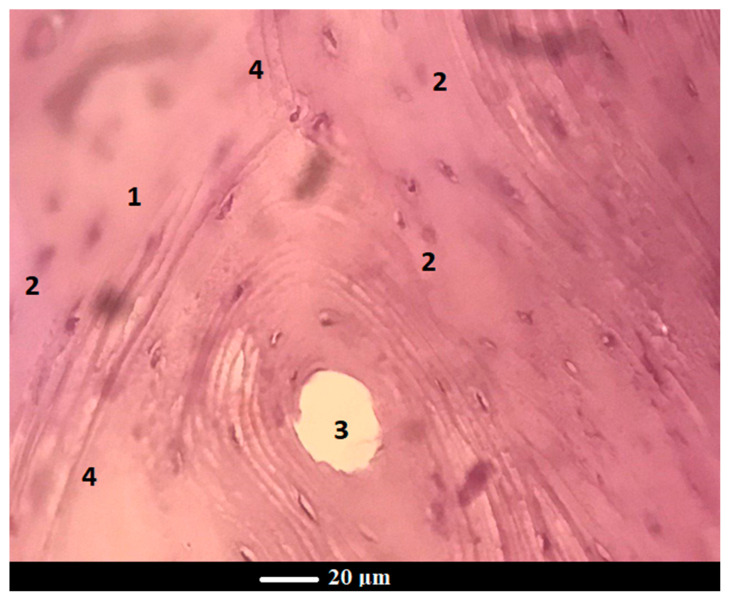
The cross-section of the femur implanted with scaffold at 6 months: normal bone tissue in the osteon’s field of view contains a blood vessel in the center and concentric bone tubules along the periphery Legend: 1—new bone, 2—osteocytes, 3—osteon, 4—cement lines.

Figure 8a displays a bone plate at the site of scaffold grafting with signs of complete regeneration and a medullary cavity occupied with bone marrow. In Figure 8b, one can see a bone structure at the glass powder implantation site after 6 months of implantation, where there are distinct boundaries of the newly-formed bone (a pale pink color) and the old bone (a reddish color) along the periphery. Apparently, with an increase in the healing time, there is an increase in the reparative/regenerative capacity of bone tissue in the defect area with scaffold grafting, which yields the formation of matured bone with the presence of abundant osteocytes and bone marrow cells. Under higher magnification, the histological examination of the scaffold-implanted defect showed the presence of mature bone at 6 months post-implantation, which was entirely composed of the Haversian systems (osteons), comprising concentric lamellae of the bone matrix surrounding a central canal and osteocytes (Figure 9). The central canal contains the vascular and nerve supplies of the osteon in the mature bone. The Haversian systems are separated from one another by cement lines, while the space between separate osteons is occupied by interstitial lamellae.

Statistical analysis was performed by using the Wilcoxon–Mann–Whitney test. The results of the calculation showed that the U values were higher than the critical tabulated U value, i.e., U calculated > U critical always (the data for calculation are reported in Table 3). This means that there is no statistically significant difference between the intensities of bone formation scores in the control and experimental groups at the two tested periods of implantation (3 and 6 months).

It is well documented that the process of implant integration is a complex biological time-consuming process that may result in undesired resorption or rejection reactions, as well as the possibility of transporting infectious agents to the host [41,42]. It was revealed that the new bone formation began in the marrow and advanced to the periphery of the defect, however, the repair did not occur uniformly with the same tissue pattern across the entire defect, and the healing of the central defect region is typically delayed [43]. Furthermore, the woven bone is remodeled into lamellar bone over the course of months to years, which allows for the restoration of the canal and its bony properties [43]. Recently-synthesized 3D porous strontium-containing BGs demonstrated to outperform Hench’s 45S5 BG with regard to bone-material contact, promoting the formation of a more mature-like lamellar bone rather than woven bone [44]. Normal healthy trabecular bone was formed as early as 6 weeks post-implantation and less residual Sr-doped BG material was found in the defect as compared to 45S5 BG-treated defects [44].

An ideal bone grafting material should safely dissolve once it has performed its function in the body; therefore, then being fully replaced by new healthy tissue [45]. The bioactive 47.5B material tested in the present study fulfills this key requirement and actually stimulates new bone regeneration at the defect site; however, this occurs regardless of the presence of a 3D macroporous architecture (scaffold). This apparently surprising result can be explained, considering that robocast 47.5B BG scaffolds exhibit a grid-like arrangement of macro-channels that do not closely mimic the trabecular architecture of cancellous bone with its peculiar morphological, topographical and mass transport properties, which have an obvious impact on new bone growth. Scaffolds that were provided with a foam-like architecture better replicated the bone microstructure and microenvironment as compared to the implants with oriented pores [22], thus further accelerating bone healing and regeneration. In this regard, an in vivo pilot study comparing gel-cast scaffolds of PSrBG (44.5SiO_2_-4Na_2_O-4K_2_O-4.5P_2_O_5_-17.8CaO-17.8SrO-7.5MgO, mol.%) and ICIE16 (49.46SiO_2_-36.6CaO-6.6Na_2_O-1.07P_2_O_5_-6.6K_2_O, mol.%) showed that the foamed morphology supports and sustains bone growth across a 12-week time frame within a femoral head defect in a rabbit model. Both scaffolds underwent complete biodegradation, and ICIE16 seemed to encourage more bone ingrowth than PSrBG after 12 weeks in situ, showing a bone morphology similar to other regions of the host femoral head [46].

These aspects deserve further investigation and motivate future research on the design of optimal scaffold geometries that are able to maximize osteointegrative and osteoinductive effects.

## 4. Conclusions

In the present study, robocasting technology allowed for the fabrication of well-reproducible silicate glass cylindrical scaffolds with grid-like porous architecture (total porosity around 50 vol.%). The scaffolds exhibited bioactive properties upon immersion in SBF, as demonstrated by the formation of a layer of calcium phosphate on the surface of pore walls. The 3D-printed bioactive glass scaffolds were then characterized in terms of their osteostimulatory capability in vivo. After 6 months of implantation in rabbit femur, residues of glass were completely embedded into bone trabeculae, being indistinguishable from the host or newly-formed bone, thus suggesting that full resorption and/or osteointegration took place. However, no statistically significant differences in new bone formation were found between the porous scaffolds and granules of the same glass used as the control. Nevertheless, histological examinations revealed that scaffold-implanted defects were associated with a more homogenous tissue formation throughout the entire defect as compared to the control. These results motivate further research on the relationship between scaffold architecture/morphology and the osteogenesis addressed to developing an optimal scaffold design for bone applications.

## Figures and Tables

**Table 1:** Protocol of implantation procedure.

Observation Stage	Control Group (C)–47.5B Powder	Experimental Group (E)–47.5B Scaffolds
3 months	4 animals per group with numbering in the range No. 1–4	4 animals per group with numbering in the range No. 5–8
6 months	4 animals per group with numbering in the range No. 9–13	4 animals per group with numbering in the range No. 14–16

**Table 2:** Geometrical characterization of robocast scaffolds.

	Mass/g	Diameter/mm	Height/mm	*ε*_0_/vol.%
Mean value	0.08	4.37	4.21	50.64
Standard deviation	0.01	0.14	0.13	4.02

**Table 3:** Bone formation score for C and E groups according to histological scoring scale (Section 2.4).

Observation Stage	(C)	(E)
3 months	n = 1/score 3 n = 2/score 2 n = 3/score 3 n = 4/score 4	n = 5/score 2 n = 6/score 2 n = 7/score 3 n = 8/score 3
6 months	n = 9/score 4 n = 10/score 3 n = 11/score 4 n = 12/score 4	n = 13/score 4 n = 14/score 4 n = 15/score 4 n = 16/score 4

## Data Availability

Data are included within this article.
